# Multi-omics reveals the key and specific miRNA-mRNA modules underlying salt tolerance in wild emmer wheat (*Triticum dicoccoides* L.)

**DOI:** 10.1186/s12864-022-08945-3

**Published:** 2022-10-25

**Authors:** Guang Yang, Wenqiu Pan, Rui Cao, Qifan Guo, Yue Cheng, Qinlong Zhao, Licao Cui, Xiaojun Nie

**Affiliations:** 1 State Key Laboratory of Crop Stress Biology in Arid Areas and College of Agronomy, Northwest A&F University, Yangling, Xianyang, 712100 Shaanxi China; 2 College of Biological Science and Engineering, Jiangxi Agricultural University, Nanchang, 330045 Jiangxi China

**Keywords:** Wild emmer, Salt stress, miRNA-mRNA module, Co-expression network

## Abstract

**Background:**

Salt stress is one of the most destructive environmental factors limiting crop growth and development. MicroRNAs (miRNAs) are a class of conserved endogenous small non-coding RNAs, playing the crucial role in regulating salt response and tolerance in plants. However, the miRNAs in wild emmer wheat, especially the key and specific salt-responsive miRNAs are not well studied.

**Results:**

Here, we performed small RNA, transcriptome, and degradome sequencing of both of salt-tolerance (ST) and salt-sensitive (SS) wild emmer genotypes to identify the miRNA-mRNA modules associating with salt tolerance. Totally, 775 miRNAs, including 361 conserved known miRNAs and 414 novel miRNAs were detected. Differential expression analysis identified 93 salt-responsive miRNAs under salt stress. Combined with RNA-seq and degradome sequencing analysis, 224 miRNA-mRNA modules displayed the complete opposite expression trends between ST and SS genotypes, most of which functionally enriched into ROS homeostasis maintaining, osmotic pressure modulating, and root growth and development. Finally, the qRT-PCR and a large-scale yeast functional screening were also performed to initially validate the expression pattern and function of candidate genes.

**Conclusions:**

This study reported the key and specific miRNA-mRNA modules associated with salt tolerance in wild emmer, which lay the foundation for improving the salt tolerance in cultivated emmer and bread wheat through miRNA engineering approach.

**Supplementary Information:**

The online version contains supplementary material available at 10.1186/s12864-022-08945-3.

## Background

Salinity is an increasing environmental stress that severely threats global agricultural production. It is estimated that approximately 20% of the irrigated soils worldwide are suffering salt stress [1]. More than 50% of the arable land worldwide will be salinized by 2050 due to unreasonable irrigation, unsustainable cultivation and climate change [2]. Meanwhile, crop production is also facing many problems, such as the rapidly growing population, reduced water and fertilizer inputs and unfavorable climate conditions. Therefore, uncovering the molecular mechanisms underlying salt tolerance is very urgent for the development of salt-resistant crops for future agricultural production.

Small RNA-mediated regulatory module or pathway play key roles on biological processes including cell growth, gene transcription and translation. MicroRNAs (miRNAs), as one class of small non-coding RNAs, are 20–24 nucleotide (nt) in size, which are derived from single-stranded stem-loop precursors, playing the negative regulators on gene expression at post-transcriptional level by promoting degradation or repressing translation of target mRNAs [3]. Extensive studies have demonstrated the importance of miRNA in plant growth and development as well as responding to abiotic stress and biotic stress. For instance, miR319 is known to target *TEOSINTE BRANCHED1/CYCLOIDEA/PCF (TCP)* transcripts and regulates leaf development, flower development, tillering and grain yield in crops [4, 5]. And some conserved salt-related miRNAs have also been reported, such as miR156, miR393, miR528 [6–8]. Moreover, more and more miRNA-mRNA modules underlying salt tolerance were also functionally validated. For example, miR172-IDS1(*INDETERMINATE SPIKELET1*) signaling module was found to confer salt tolerance through maintaining ROS homeostasis in rice and wheat [9]. In rice, miR528-AO (l-ascorbate oxidase) module could modulate the ascorbic acid and abscisic acid metabolism as well as ROS scavenging to enhance salt tolerance [8]. The miR156/SPL module played the crucial in regulating salt stress tolerance through activating the expression of *MdWRKY100* in apple [6].

With the development of high-throughput sequencing technologies, small RNA sequencing combined with degradome sequencing have been widely used to identify miRNA and its splicing targets in various plants at the genome-wide scale. Yang et al.integrated transcriptome, small RNAs and degradome sequencing of the NaCl-free and NaCl-treated sweet potato to identify 314 salt-related miRNA and 636 target genes, uncovering a key regulatory network and providing insights into salt-tolerance in sweet potato [10]. Similarly, through combined analysis of small RNA, transcriptome, and degradome sequencing of two genotypes with contrasting salt resistance in sesame, 21 key miRNA-mRNA pairs with opposite expression trends between salt-resistant genotype and salt-sensitive genotype were obtained [11].

Wild emmer wheat (*Triticum dicoccoide*s. L, AABB) is the direct progenitor of cultivated emmer wheat and bread wheat, which is originated from the Fertile Crescent and adapts to a broad range of environments with rich genetic alleles related to abiotic stress [12]. Some genotypes of wild emmer were found to be highly tolerant to salt stress, providing the indispensable reservoir of genetic diversity for breeding salt tolerant wheat to ensure productive and stable wheat production [13]. However, the key and species-specific miRNA, especially the miRNA-mRNA modules underlying salt tolerance in wild emmer is not well studied up to now. In this study, a combination of small RNA sequencing, transcriptome sequencing and degradome sequencing of the salt-tolerance genotype A5 and salt-sensitive genotype C2 under salt treatment of 0.5 h, 3 h, 8 h, 27 h was performed to identify salt-responsive miRNAs as well as the miRNA-mRNA modules in wild emmer wheat, which not only enriched the genetic resource for salt tolerance, but also lay the foundation to genetic improvement of the salt tolerance in cultivated emmer wheat and bread wheat through miRNA bioengineering.

## Results

### Small RNA Sequencing and Identification of Known and Novel miRNA

To obtain the key and specific salt-responsive miRNAs in wild emmer, 48 small RNA libraries of ST and SS genotype were constructed at 0.5 h, 3 h, 8 h and 27 h after 150 mM NaCl treatment. After small RNA sequencing, a total of 1438.33 million reads were generated (Table 1). Of these, approximately 697.69 million reads with length < 18 nt or > 25 nt and with 3’ adaptor sequence was discarded, followed by removal of ribosomal RNA (rRNA), transfer RNA (tRNA), small nucleolar RNA (snoRNA) and repeat sequences. The length distribution of reads of unique sRNAs indicated that 24 nt (18.12%) were the most abundant, followed by 21 nt (16.32%), 22 nt (13.19%) and 20 nt (12.42%).

**Table 1:** Summary of small RNA, Degradome and Transcriptome sequencing reads

statistic of sequencing data	million
**small RNA sequencing**	
raw reads	1438.33
3ADT&length (< 18nt and > 25nt) filtered	697.69
miRNA reads	326.67
rRNA reads	90.13
tRNA reads	99.23
snoRNA/snRNA reads	1.33
repeat reads	1.34
**degradome sequencing**	
raw reads	91.06
reads < 15nt after removing 3' adaptors	0.48
mapped reads	70.10
unique mapped reads	12.14
**transcriptome sequencing**	
raw reads	3186.069
mapped reads	2880.15
unique mapped reads	1966.56

**Fig. 1:**
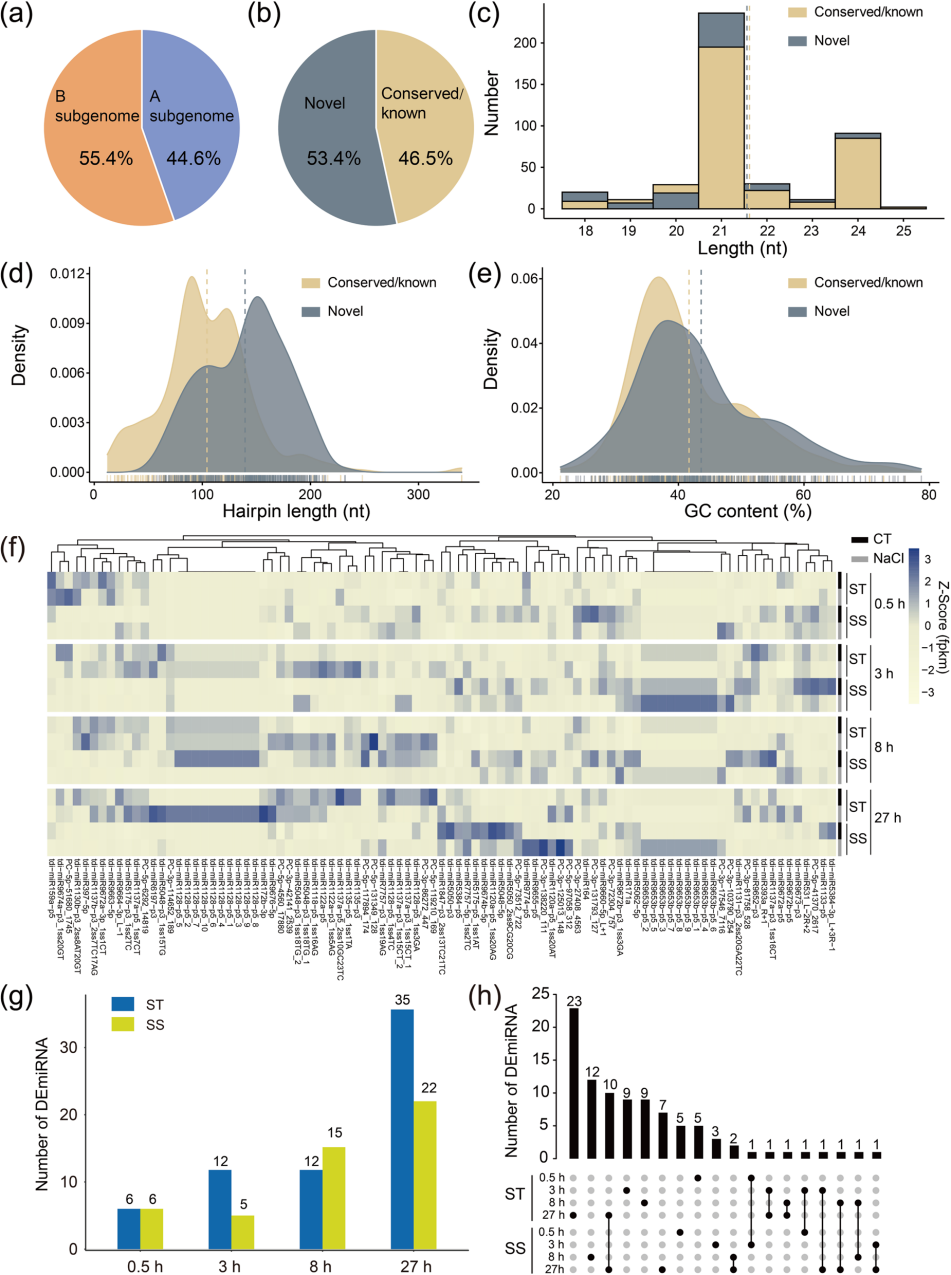
Characteristic and expression profile of identified miRNAs in wild emmer wheat. **(a)** Distribution of all miRNAs on the subgenomes. **(b)** The percentage of the identified novel and conserved miRNAs. **(c)** Length distribution of all miRNAs. **(d)** Hairpin length distribution of all miRNAs. **(e)** Distribution of nucleotide GC content of all miRNAs. **(f)** Expression profile of all DEmiRNAs. **(g)** Number of DEmiRNAs at each treatment time point of ST and SS genotype. **(h)** Intersection of DEmiRNAs in different genotypes and different time points. CT: control without NaCl treatment; NaCl: 150 mM NaCl treatment. ST: salt tolerance genotype; SS: salt sensitive genotype

Though searching against the miRbase 22.0, a total of 775 unique miRNA were identified from all samples, including 361 conserved/known and 414 novel miRNAs (Fig. 1a, b; Additional file 1: Table S1). Among them, the known miRNAs belonged to 34 miRNA families. The largest family was miR1120, followed by miR1122 and miR818. The length of all identified miRNAs ranged from 18 to 25 nt and 21 nt was the most abundant, followed by 24 nt (Fig. 1c). The hairpin length of all miRNAs was mainly concentrated in 100–120 nt (Fig. 1d). The average GC content of these miRNAs was 42.74% (Fig. 1e).

### Identification of the salt-responsive miRNAs

Based on the expression level**,** a total of 456 miRNAs (conserved/known: 234; novel: 222) with middle or high expression levels were retained for further analysis, of which 68 miRNAs were specifically expressed in ST genotype and 76 were specifically expressed in SS. Then, the differentially expressed miRNAs (DEmiRNAs) were identified between NaCl and control group at different time points (Fig. 1f; Additional file 1: Table S2). Under NaCl treatment, 63 DEmiRNAs were identified in the ST genotype, of which 2 DEmiRNAs were shared by different time points (3 h *vs.* 27 h and 8 h *vs.* 27 h). While 45 DEmiRNAs were identified in the SS genotype, of which 3 DEmiRNAs were shared by different time points (one was shared by 3 h *vs.* 27 h and two were shared by 8 h *vs.* 27 h). Specifically, there were 6 (3 up-regulated and 3 down-regulated) DEmiRNAs after 0.5 h of treatment in the ST genotype and the number of DEmiRNAs were markedly increased at 3 h (6 up-regulated and 6 down-regulated), then reached a maximum at 27 h (16 up-regulated and 19 down-regulated) (Fig. 1g). Similar to the ST genotype, there was also the least amount of DEmiRNAs at 0.5 h (3 up-regulated and 3 down-regulated) and the largest amount at 27 h (13 up-regulated and 9 down-regulated) in the SS genotype. However, the number of DEmiRNAs did not change significantly at 3 h (5 down-regulated) to 8 h (6 up-regulated and 9 down-regulated). Overall, there were a little DEmiRNAs shared between ST and SS, and the most common DEmiRNAs were occurred at 27 h after treatment (Fig. 1h). These results indicated that miRNAs displayed the obvious genotype- and stage-specific patterns when subjected to salt stress in wild emmer.

### Identification of targets for DEmiRNAs by degradome sequencing

Furthermore, degradome sequencing was applied to find the target genes of the identified DEmiRNAs. A total of 90.58 million clean reads were used to identify cleavage sites (Table 1). After in silico analysis, 13,048 transcripts of 6,479 genes were identified as targets for 92 DEmiRNAs (Additional file 1: Table S3), of which 15,762 DEmiRNA-mRNA pairs (Different cleavage nucleotide positions between the same miRNA and mRNA were considered to be different pairs) were obtained. Among them, 6,777 and 5,488 genotype-specific DEmiRNA-mRNA pairs were identified for ST and SS genotype respectively, and 3,497 pairs were shared by ST and SS genotype (Additional file 1: Table S3). All of the DEmiRNAs, except tdi-miR1128-p5_1ss3GA, could regulate more than one target, indicating the diverse roles of miRNA may play in involving in salt stress response. Moreover, 2,047 (15.7%) targets could be cleaved by more than one miRNA. For example, TRIDC5BG065490.1, encoding Glutathione S-transferase, was found to be simultaneously targeted by PC-5p-4560_17880, tdi-miR5384-p5 and tdi-miR9674a-p3_1ss20GT. Subsequently, 462 transcription factors were identified as the targets of DEmiRNAs, including 33 NACs, 29 bHLHs and 26 bZIPs. GO enrichment analysis found that the 13,048 targets were significantly enriched into 174 GO terms and most of the genes were assigned to biological process (92), followed by molecular function (53) and cellular component (29). The most significant enriched GO term in the cellular component was chloroplast (GO:0,009,507, 1.77E-238), followed by plasma membrane (GO:0,005,886, 1.10E-152), cytoplasm (GO:0,005,737, 4.51E-140). The most significant enriched GO term of biological process was oxidation–reduction process (GO:0,055,114, 6.70E-48), followed by protein phosphorylation (GO:0,006,468, 1.46E-33) and response to salt stress (GO:0,009,651, 6.66E-26). The top three GO term of molecular function was protein binding (GO:0,005,515, 1.21E-207), ATP binding (GO:0,005,524, 3.96E-47) and ATPase activity (GO:0,016,887, 4.39E-23) (Additional file 2: Figure S1).

### Identification of DEmiRNA-DEmRNA Modules

To comprehensively obtain the miRNA-mRNA modules underlying salt tolerance in wild emmer, RNA-seq analysis of the same 48 samples for small RNA sequencing were performed. A total of 3186.069 million raw reads were generated and 2880.15 million clean reads were mapped on wild emmer reference genome (Table 1) and 67,375 genes and 300,710 transcripts were assembled for downstream analysis. By calculating gene expression level, 136,212 transcripts were found to be expressed (the FPKM of genes was larger than 1 in at least one of all samples). Then, a total of 38,282 unique transcripts that differentially expressed at least one treatment time point were identified. Of these, 12,062 and 16,064 were specifically identified in ST and SS genotype, respectively (Additional file 1: Table S4).

Generally, the change in miRNA and mRNA expression was in the opposite direction [14]. By integrating the results of small RNA, degradome and transcriptome sequencing, 389 and 303 coherent DEmiRNA-DEmRNA pairs (degradome reads support; opposite expression trend between miRNA and mRNA; differential expression occurred at the same time point) were identified in ST and SS genotype, respectively (Additional file 1: Table S5). Of these, 17, 30, 16 and 326 coherent pairs were identified at 0.5 h, 3 h, 8 h and 27 h of ST genotype, while the SS genotype had 11, 36, 49 and 207 coherent pairs at 0.5 h, 3 h, 8 h and 27 h, respectively.

**Fig. 2:**
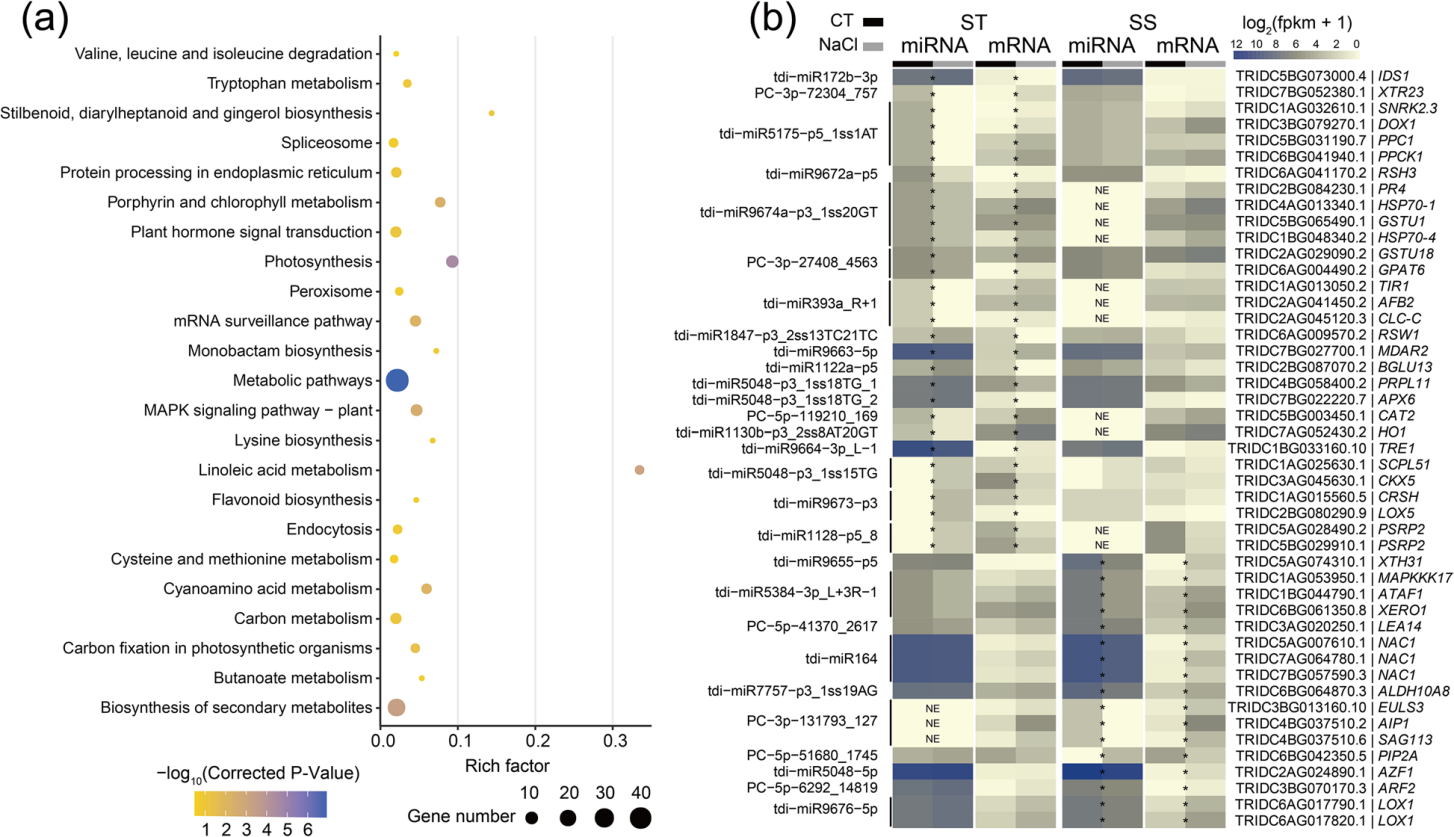
Expression profile of salt tolerance-related DEmiRNA-DEmRNA pairs and KEGG enrichment of all candidate targets. **(a)** KEGG enrichment analysis of target genes of candidate salt tolerance-related DEmiRNA-DEmRNA pairs. **(b)** Expression profile of 47 DEmiRNA-DEmRNA pairs which target genes had orthologues with validated salt tolerance function. ST: salt tolerance genotype; SS: salt sensitive genotype; CT: control without NaCl treatment; NaCl: 150 mM NaCl treatment; *: significantly differentially expressed after treatment; NE: no expression

To further discover the key candidates for salt tolerance, we selected the coherent pairs displaying the differential expression trend between ST and SS genotypes, namely the modules whose miRNA and mRNA were significantly down-regulated or up-regulated in ST, but little changed (expression levels did not change significantly after salt treatment) or not found in SS, or displayed little changed or not found in ST but down-regulated or up-regulated in SS genotype was considered as the key candidate modules underlying salt tolerance. Totally, 54 DEmiRNAs met the criteria and 224 unique coherent DEmiRNA-DEmRNA pairs were obtained as the candidates (Additional file 1: Table S6). Out of them, 88 miRNA-mRNA coherent pairs were significantly differential expressed in the ST but little changed in SS genotype. Accordingly, 90 pairs were significantly differential expressed in SS but little changed in ST genotype. Otherwise, 32 and 14 coherent pairs were specifically found in ST and SS genotype, respectively. GO enrichment showed that these target genes mainly involved in the salt tolerance-related terms, such as response to abscisic acid, oxidoreductase activity and root development (Additional file 2: Figure S2). KEGG enrichment also found they mainly involved in salt-related pathways, such as MAPK signaling pathway, plant hormone signal transduction, photosynthesis. (Fig. 2a).

To provide some insight into their function, we investigated the orthologues of target genes in candidate DEmiRNA-DEmRNA modules based on Arabidopsis and rice genes. Results showed that 203 targets had orthologues with Arabidopsis or rice, of which 47 genes have been reported to be related to salt stress by previous studies (Fig. 2b; Additional file 1: Table S6). For instance, *IDS1*, an AP2/ERF transcriptional factor that is related to salt tolerance [9], was targeted by tdi-miR172b-3p and down-regulate at 0.5 h in ST genotype. At 3 h after treatment, tdi-miR393a_R + 1, targeting TRANSPORT INHIBITOR1/AUXIN-SIGNALLING F-BOX (*TIR1/AFB2)*, was down-regulated in ST but not found in SS; tdi-miR1128-p5_8 targeting both 30S ribosomal protein (TRIDC5AG028490.2 and TRIDC5BG029910.1) that associated with salt tolerance. *APX*, an L-ascorbate peroxidase that is related to ABA and to indole-3-acetic acid, was targeted by tdi-miR5048-p3_1ss18TG_2 and down-regulated at 8 h after treatment. In SS genotype, *AZF1* related to salt sensitivity was targeted by tdi-miR5048-5p and up-regulated at 27 h.

**Fig. 3:**
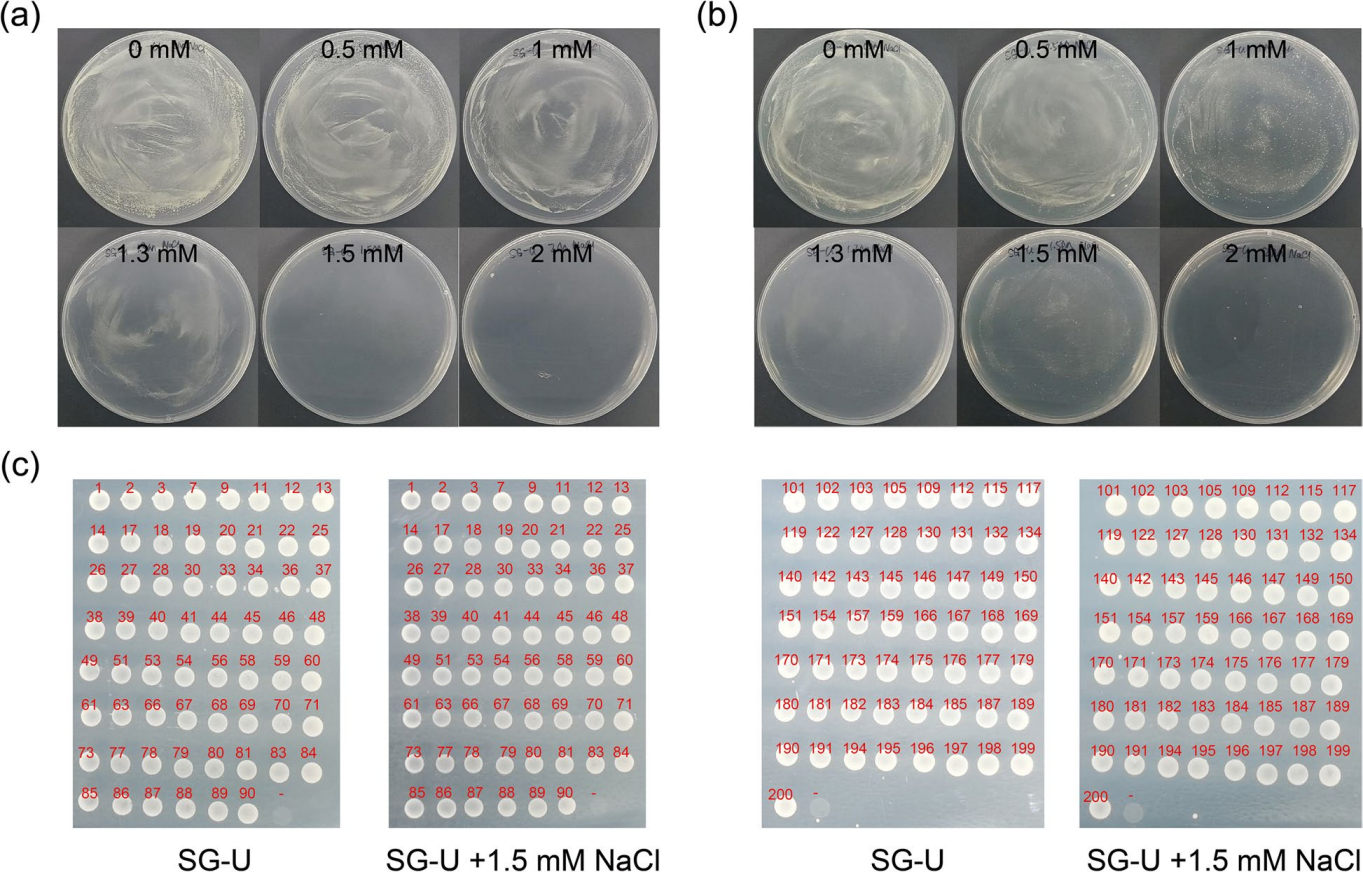
Yeast functional screening of the full-length cDNA library of ST genotype. **(a-b)** Yeast transformed with empty vector and salt-stressed *T. dicoccoides* cDNAs grown in SG-Ura plates with 0, 0.5, 1, 1.3, 1.5 and 2 mM NaCl respectively. **(c)** Re-spotted of 119 successfully cultured salt tolerance-related clones in SG-Ura plates with 1.5 mM NaCl. Each number represented an individual clone

Finally, we further filtered the candidate genes underlying salt tolerance through large-scale yeast functional screening of the full-length cDNA library of ST genotype (see Materials and methods for more details) (Additional file 2: Figure S3; Fig. 3a, b). Then, 200 clones displaying salt tolerance were randomly selected to perform Sanger sequencing (Fig. 3c), of which 4 four full-length cDNAs showed complete sequence consistence with the mRNAs in these DEmiRNA-DEmRNA modules underlying salt tolerance, including TRIDC1AG054250.1 (*LHB1B2*) targeting by tdi-miR9652-p3, TRIDC2BG049890.4 (*GAPA-2*) targeting by tdi-miR5048-p3_1ss15TG, TRIDC2AG072020.2 (*RD21A*) targeting by tdi-miR9664-3p_L-1, TRIDC5AG008340.1 targeting by tdi-miR9673-p3, which further demonstrated the reliability of our results. The key and genotype-specific DEmiRNA-DEmRNA modules enriched the miRNA resource for salt tolerance and also provided the potential candidates for further functional analysis.

### WGCNA Network of DEmiRNA-DEmRNA modules underlying salt tolerance

**Fig. 4:**
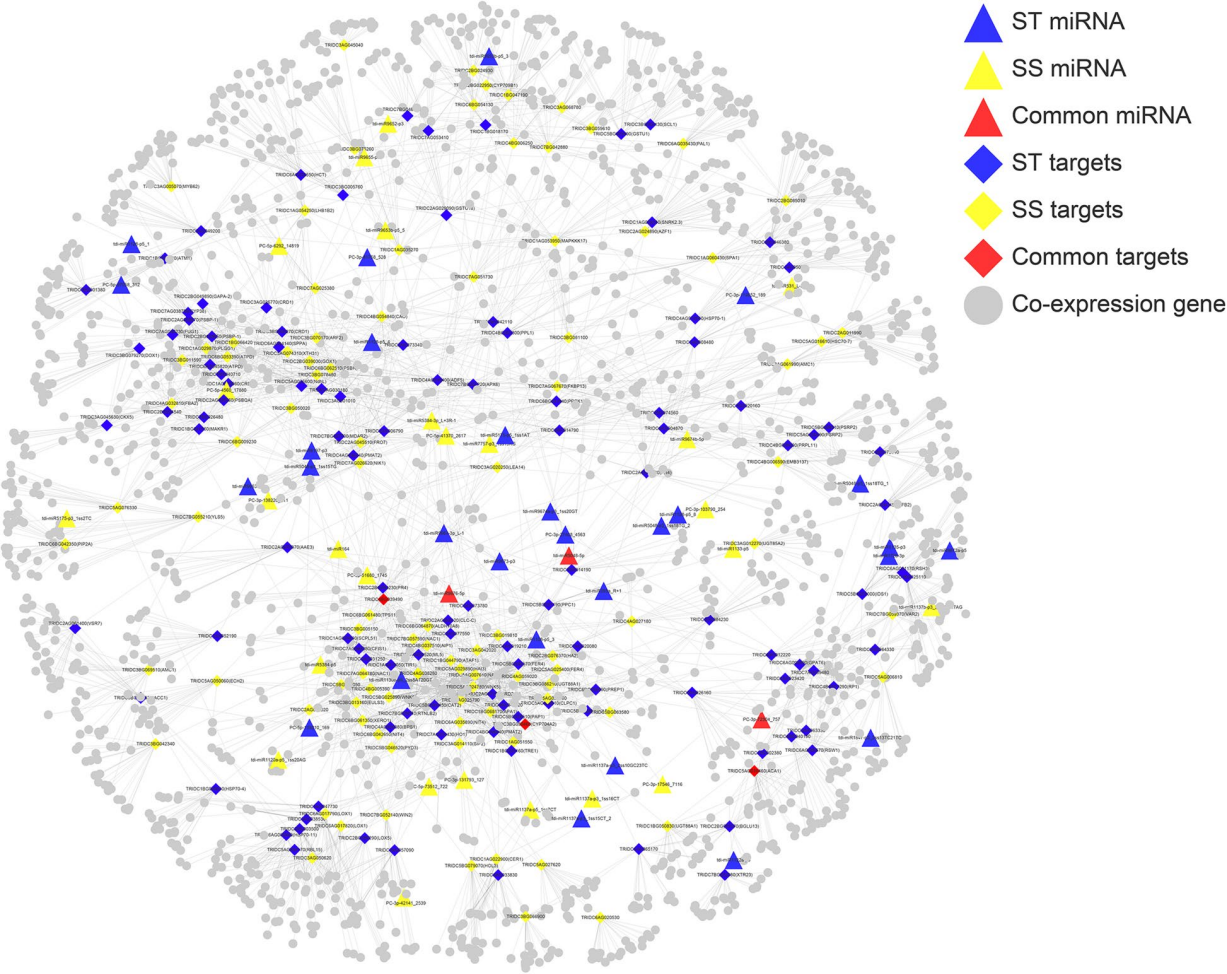
Network of salt tolerance-related DEmiRNA-DEmRNA pairs and top 20 co-expressed genes. Blue color represented ST genotype; yellow color represented SS genotype; red color represented the common loci of ST and SS genotype. Triangle represented miRNAs; diamond represented target genes; grey represented co-expression gene

In order to further expand the functional relationship of DEmiRNA-DEmRNA modules underlying salt tolerance, we constructed the co-expression network based on all expressed genes and the top 20 co-expressed genes with connectivity to candidate targets were filtered (Fig. 4; Additional file 1: Table S7). GO enrichment found that co-expressed genes mainly enriched into the terms related to salt response, such as response to salt stress (GO:0,009,651), response to oxidative stress (GO:0,006,979), response to abscisic acid (GO:0,009,737), response to osmotic stress (GO:0,006,970) (Additional file 1: Table S8). For instance, *SOS1* gene was found to be co-expressed with the target gene (*HCT*) of tdi-miR5048-p3_1ss15TG in ST genotype; the auxin signaling gene, *ARF8*, was also identified to be co-expressed with tdi-miR393a_R + 1/AFB2 module. The results of co-expression network further suggested that candidate targets played important roles in response to salt stress.

### Validation of mRNA and miRNA Expression by qRT-PCR

Then, the six candidate DEmiRNA-DEmRNA pairs were randomly selected for qRT-PCR analysis to validate their expression. qRT-PCR results showed good consistence with the sequencing results, which confirmed the expression trend of the six candidate DEmiRNA-DEmRNA pairs. In detail, tdi-miR172-3p was up-regulated at 0.5 h after treatment in ST and SS genotype, and its target was significantly down-regulated in ST genotype while displayed no differential expression in SS genotype. Although tdi-miR5175-p5_1ss1AT/SnRK2.3 module was not differentially expressed through qRT-PCR validation, the expression tread of this module was consistent with small RNA-seq and RNA-seq in ST genotype. In addition, the expression levels of tdi-miR9674a-p3_1ss20GT and its two target genes (*GSTU1* and *HSP70-4*) at 3 h were also verified to be consistent with the sequencing data. Moreover, two novel miRNA, PC-5p-51680_1745 and PC-5p-6292_14819, was up-regulated and down-regulated at 8 h and 27 h in SS genotype respectively, and their targets showed the opposite expression trends (Additional file 1: Table S6; Additional file 2: Figure S4).

## Discussion

MiRNAs are a class of small non-coding RNAs playing the crucial regulatory roles in gene expression at the post-transcriptional level, which also provide the effective candidates for crop improvement [14]. Recently, a few studies have been carried out in identifying or comparatively profiling salt tolerance-related miRNAs and target genes in crops [9–11, 15, 16]. High salinity is one of the major limiting factors for bread wheat production. As the genetic reservoir of bread wheat, wild emmer wheat has excellent salt tolerance and it is natural miRNA toolboxes in response to stress [17], but it remains unclear how miRNAs involve in salt tolerance in wild emmer wheat. In this study, we performed a comprehensive analysis of small RNA, degradome and transcriptome sequencing of the salt tolerant and sensitive wild emmer genotypes at 0.5 h, 3 h, 8 h and 27 h after NaCl treatment. Based on the strict criteria for annotation of plant miRNAs, 775 miRNAs were identified, including 414 novel miRNAs, which significantly enriched the genetic resources of miRNA in wild emmer wheat. The features of identified miRNAs like the length of mature miRNA, hairpin length, and GC content were in concordance with the previous studies [9, 10, 15, 16]. Furthermore, 93 DEmiRNAs were identified while a few miRNAs were shared by different time points, showing the difference in salt stress response mechanism at each time point. And the number of DEmiRNAs in the ST genotype started to increase significantly at 3 h after treatment, while the SS genotype was at 8 h after treatment, indicating that ST genotype responded faster than SS genotype to salt stress. Moreover, 78 DEmiRNAs were genotype-specific under NaCl treatment, indicating that these miRNAs may have contributed to the phenotypic differences in salt tolerance between ST and SS genotype. Our study also found some salt-related conserved miRNAs, such as miR172 and miR393 [7, 9]. MiR172 has been reported to be induced by salt stress in rice, and overexpression of miR172 in rice and bread wheat can enhance the salt tolerance [9]. Similar to the expression profile in rice, miR172 in our results was also upregulated at 0.5 h after treatment in the ST genotype. MiR393 was significantly downregulated in sesame, maize and cotton [11, 18, 19]. In our results, we also found that miR393 showed down-regulation after NaCl treatment. Overall, our results identified some genotype-specific and also some conserved salt tolerance-related miRNAs, of which the conserved miRNAs are probably responsible for controlling the basic response and developmental processes under salt stress, while the genotype-specific miRNAs are involved in the specific regulation and functions on different genotype.

**Fig. 5:**
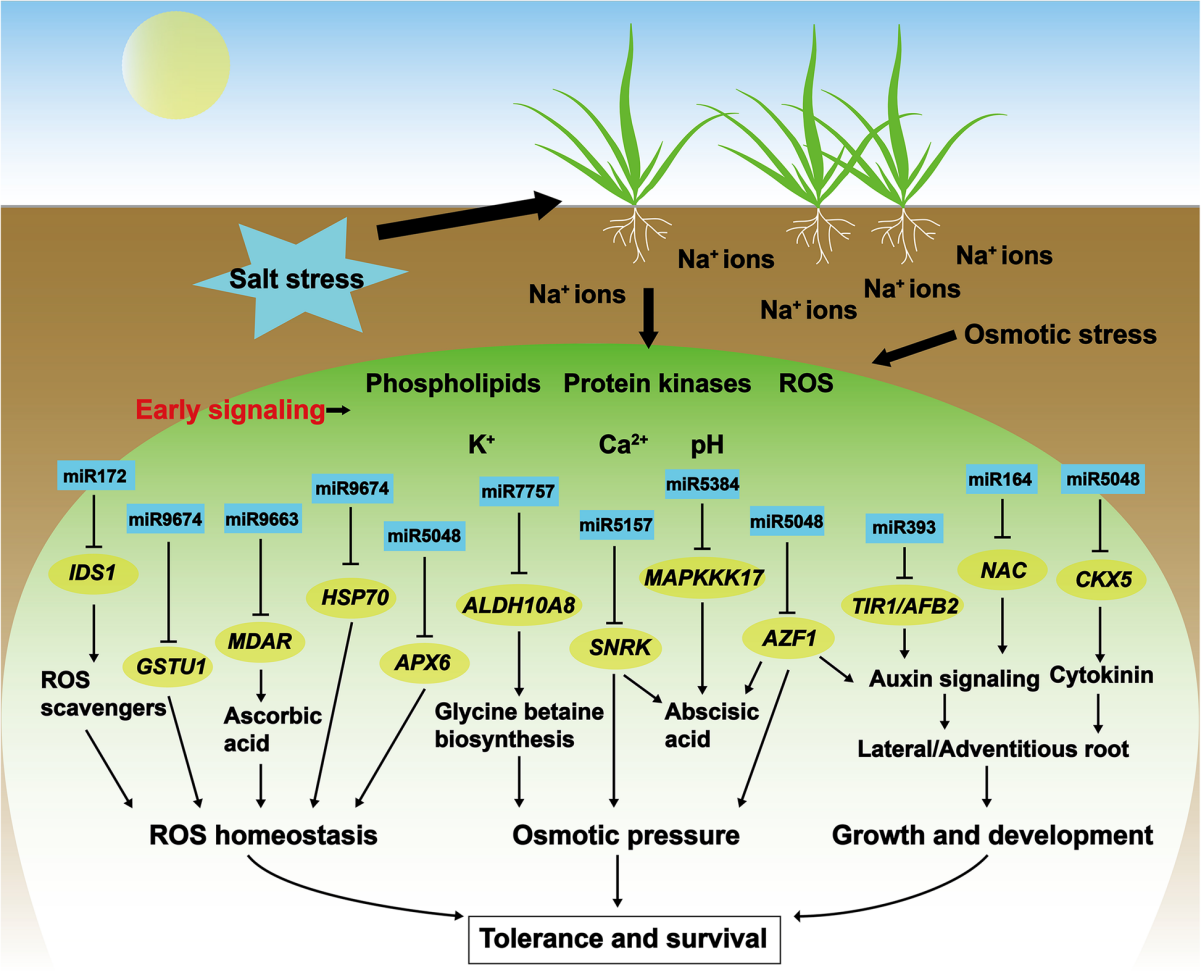
A model illustrating the mechanism of miRNA function on salt tolerance in wild emmer wheat

To further clarify the regulatory functions of DEmiRNAs under salt stress, we used degradome and transcriptome sequencing to detect the targets of miRNAs. Only one of the DEmiRNAs did not identify target genes, which may be due to the low expression level. Consistent with previous studies [10, 11, 15, 16], most of DEmiRNAs can target more than one target and the same target can be cleaved by multiple miRNAs. Generally, the up-regulation of miRNA leads to the down-regulation of their target genes and vice versa. Since the differential expression of specific genes and non-coding RNA between salt-tolerant and sensitive genotypes under salt stress conditions is one of the main reasons for the difference in salt tolerance between the two lines [20], we focused on miRNA-mRNA pairs with significant differences between ST and SS genotype. According to the different molecular mechanisms of salt tolerance, DEmiRNA-DEmRNA pairs can be mainly classified into three categories, including reactive oxygen species (ROS) scavenging, growth and developmental responses, and osmotic pressure regulation (Fig. 5). Salinity stress causes planta accumulation of ROS, which can result in oxidative stress and cell damage [21]. Salt-tolerant plants can scavenge ROS through different pathways and maintain the balance between ROS generation and scavenging. ROS-scavenging genes are key factors in modulating ROS homeostasis. One transcriptional repression gene of ROS-scavenging genes, *IDS1*, was reported to be targeted by miR172 [9]. As a crucial molecular rheostat in maintaining ROS homeostasis, miR172/*IDS1* module was also demonstrated to be related to salt tolerance in bread wheat and rice. Our results also found the miR172/*IDS1* module in the ST genotype, but not in SS. The expression of miR172 was upregulated after NaCl treatment and *IDS1* was downregulated, showing the potentially conserved function in cereal crops. Moreover, we also found novel miRNA-mRNA modules involved in ROS signal modulating, of which target genes have been reported to be related to salt tolerance, containing miR9663/*MDAR2*, miR5048/*APX6*, miR9674/*GSTU1*, miR9674/HSP70. Ascorbate (AsA) is a major antioxidant and free-radical scavenger in plants [22]. As the target gene of miR9663, *MDAR2* is an important enzyme of the AsA-glutathione (GSH) cycle and involved in salt tolerance through scavenging of ROS [23]. Overexpressing lines of tobacco also confirmed that *MDAR* can enhance tolerance against salt stress [24]. Target gene of miR5048, Cytosolic ASCORBATE PEROXIDASE6 (APX6) (coding for the hydrogen peroxide-scavenging enzyme), also modulates the ROS signal and the Arabidopsis that lacking APX6 accumulate higher levels of ROS [25]. Moreover, GSTU, a kind of plant-specific glutathione transferases, influences the redox state of GSH and AsA. GSTU mutant of Arabidopsis induced the accumulation of ROS and showed the compensating role of AsA, GSH, dehydroascorbate reductase and glutathione reductase [26]. In our results, *GSTU1* was found to be the cleavage site of miR9674 and this gene was significantly up-regulated in ST genotype after NaCl treatment but not in SS genotype, supporting the key function in response to high salinity stress. In addition, the *mtHSC70-1* knockout plants have increased levels of ROS [27]. Our results also showed that *HSP70* was targeted by miR9674 and upregulated in the ST genotype, reflecting a stronger modulating for ROS. These novel modules also indicated that the miRNA of ST genotype widely participates in the regulation of ROS homeostasis by upregulating or downregulating target genes in response to salt stress.

Salt stress in the soil also leads to osmotic stress, which causes water deficit and oxidative stress [21]. Glycine betaine is a quaternary ammonium compound that plays an important role in tolerance to abiotic stress and accumulates to osmotically significant levels in many salt-tolerant plants [28, 29]. Here, a betaine aldehyde dehydrogenase *ALDH10A8* that oxidizes betaine aldehyde to glycine betaine was identified to be the target gene of miR7757. The expression of *ALDH10A8* was upregulated in the SS genotype after treatment. According to a previous study, a balanced expression of *ALDH10A8* is important for salt tolerance and seedings in Arabidopsis [30]. Therefore, the little change of *ALDH10A8* in the ST genotype after NaCl treatment may support this conclusion. The sucrose nonfermenting1–related protein kinase 2 (*SNRK2*) was reported to have evolved specifically for hyperosmotic stress signaling and that members have acquired distinct regulatory properties [31]. SnRK family members have also been demonstrated to play crucial roles in salt stress response [32, 33]. In this study, we found that miR5175 targeted *SnRK2.3* at 0.5 h in ST genotype, indicating that it may be involved in the process of ST genotype rapid response to high salinity. In Arabidopsis, *AZF1*, a Cys2/His2 Zinc-Finger protein, was induced by osmotic stress and the overexpression of *AZF1* indicated the inhibition of plant growth under abiotic stress [34]. Furthermore, we found that *AZF1* was targeted by miR5048 in the SS genotype and significantly upregulated after NaCl treatment, but not changed in the ST genotype. Moreover, miR5048 cleaved *APX6* and *AZF1* in ST and SS genotype simultaneously, indicating the same miRNA may involve different pathways in different salt-tolerance genotypes. We speculate that this is due to genetic variation between two genotypes. In addition to the response to osmotic pressure, miR5157, miR5048 and miR5384 (cleaved *MAPKKK17*) may also be involved in the abscisic acid (ABA) signaling pathway based on the function of their targets [35].

During growth and developmental responses, roots are the main organ of plants in response to high salinity and osmotic pressure [21]. The change in root related traits will directly affect the growth and development of plants. Auxin signaling is an important factor in regulating plant growth. AUXIN RESPONSE FACTOR (ARF) is the key factor for the regulation of auxin-dependent genes. ARF can interplay with auxin/indole acetic acid repressors (*Aux/IAAs*) and these repressors form co-receptor complexes with TRANSPORT INHIBITOR1/ AUXIN-SIGNALLING F-BOX (*TIR1/AFB*) proteins to modulate jasmonic acid homeostasis and control adventitious root initiation [36]. Thus, *TIR1/AFB* is directly related to the regulation of auxin-dependent genes. Several studies have reported that *miR393–TIR1/AFB2* module has multiple functions about manipulating the auxin response. Our results found that *TIR1* and *AFB2* were targeted by miR393, and miR393 downregulated in ST but not found in SS genotype, showing the contribution of root development to the salt tolerance of ST genotype. MiR164/*NAC*, a conserved module, has been previously reported in response to drought stress and root growth [37, 38]. The *AtNAC1* has been demonstrated to enhance the growth of lateral roots [39]. However, few studies reported the salt tolerance mechanism of miR164. In our results, miR164 was downregulated in SS genotype, but not in ST. Three upregulated NAC genes were identified as the targets of miR164. The expression of miR164/*NAC* in the ST genotype did not change significantly, which may be due to the different mechanism between wild emmer wheat and other species, and further study is needed. Moreover, *AZF1* has also been shown to repress auxin signaling pathway related genes [34], indicating miR5048 may also involve root growth under salt stress. Interestingly, miR5048 also targeted a cytokinin dehydrogenase (*CKX5*) which can respond to salt stress by controlling the concentration of cytokinin to regulate plant development [40]. Therefore, miR5048 may be a key regulator of wild emmer wheat response to salt stress. Although some miRNA-mRNA modules and target genes have been reported, the function of many novel miRNAs and targets needs further study. To primarily verify the functions of the key candidate miRNA-mRNA modules, we also performed qRT-PCR and a large-scale yeast functional screening, and the results showed that verified candidate loci were consistent with multi-omics data, indicating the reliability of our results.

Previous studies have found that the key genetic variations (SNP or INDEL) can be identified in both coding genes and non-coding genes at the population level, and these variation sites could directly affect gene function. Wild emmer wheat is a wild species of tetraploid wheat, and its genetic diversity is significantly higher than that of durum wheat with some excellent variations [12]. After domestication and improvement, the durum wheat population will also have more genetic variations that meet human’s needs. For instance, *TdHMA3-B1*, encoding a metal transporter, had a non-functional variant causing high accumulation of cadmium in grain. The high-cadmium allele of *TdHMA3-B1* was identified in durum cultivars but undetected in wild emmer accessions [41]. In this study, we only compared and analyzed the miRNAs and their target genes in wild emmer wheat salt-tolerant and salt-sensitive lines respectively, and we found that the same miRNA and miRNA-mRNA modules in different genotypes showed different expression patterns. These results can be explained on the one hand by the effect of genetic variation on miRNA, on the other hand by the effect of multiple miRNA isoforms generated during miRNA processing and maturation [42]. Previous studies have shown that a single miRNA locus can generate different miRNA isoforms (isomiRs) due to post-transcriptional modification, imprecise cleavage of DCL1, etc., and these isomiRs often have different functions in response to plant development and stress [42, 43]. Further research on the effects of the genetic variations in miRNAs and targets as well as the role of isomiRs in response to salt stress could enrich the genetic basis underlying salt tolerance in wild emmer wheat, which will help to genetic improvement and breeding for salt tolerance in wheat and beyond.

## Conclusions

In summary, we profiled the salt-responsive miRNAs and their targets through a combination of small RNA, degradome and transcriptome sequencing of ST and SS genotype in wild emmer wheat in this study and the key and species-specific microRNA-mRNA modules underlying salt tolerance were identified in wild emmer. This is the first study to mine salt-responsive miRNAs in wild emmer, which lies the foundation to reveal the mechanism of salt tolerance and also contribute to future use of wild emmer to improve salt tolerance in cultivated emmer and bread wheat.

## Methods

### Plant materials and salt stress treatments

Two wild emmer genotypes with contrasting salt tolerance A5(salt-tolerance, ST) and C2 (salt-sensitive, SS), reported by previous study were used in this study [13]. All seedlings of two genotypes were hydroponically cultured and the conditions was consistent with the temperature 30 / 20 °C (day / night), humidity 55–65%, light condition 14 h (6 model) / 10 h dark (0 model). And then the 14 days-old seedlings of them were subjected to salt stress with Hoagland’s solution containing 150 mM NaCl and the normal Hoagland’s solution was used as control. The whole seedling samples were collected at 0.5 h, 3 h, 8 h and 27 h after stress treatment with three biological replicates. Total RNA was isolated from each sample of ST and SS genotype using the TRIzol reagent (Thermo Fisher Scientific, USA) according to the manufacturer’s instructions.

### Small RNA library construction, sequencing and analysis

Total RNA was purified using a TRK-1001 total RNA purification kit (LC Science, USA). Each small RNA sequencing library was constructed using TruSeq Small RNA Sample Prep Kit (Illumina, USA). Then, all libraries were used for single-end sequencing on the Illumina Hiseq2500 platform at the LC-BIO (Hangzhou, China) following the vendor’s recommended protocol. After quality control, the clean data were analyzed using the software package ACGT101-miR-v4.2 (LC Sciences, USA) with the wild emmer genome sequence as reference (http://plants.ensembl.org/Triticum_dicoccoides/Info/Index) [44]. The mapped small RNA tags were BLASTN search against miRbase 22.0 (http://www.mirbase.org) [45] to identify the conserved/known and novel 5p- or 3p- derived miRNA candidates with a mismatch of one or two nucleotide bases. Then, Mireap software (http://sourceforge.net/projects/mireap/) was used for identifying novel miRNA candidates. The differentially expressed miRNAs (DEmiRNAs) were identified by using a student’ t-test based on the normalized deep-sequencing counts with the significance threshold of 0.05.

### Transcriptome library construction, sequencing and analysis

Transcriptome sequencing of all samples used for small RNA sequencing was performed using an Illumina Novaseq™ 6000 platform at the LC-BIO (Hangzhou, China). The raw data was qualified using FastQC v0.11.7 and the primer/adaptor contamination and low-quality reads were removed using Trimmomatic v0.36 [46, 47]. Then, the obtained clean reads were mapped to the wild emmer reference genome using HISAT2 v2.1.0 [48]. Transcript and gene assembly were performed by StringTie v2.0.4 [49]. Reads were counted and assigned to transcripts using featureCounts v2.0.1 [50]. The FPKM (fragments per kilobase of transcript per million fragments mapped) was calculated using the FPKM function from the edgeR package (v 3.12.1) [51]. Transcripts with an FPKM more than or equal to 1 were considered as expressed transcript. Differentially expressed gene was identified using edgeR with the threshold of *p*-value < 0.05 and log_2_(fold change) > 1.

### Degradome sequencing and target identification

Total RNAs were quantified and purified using the TRK-1001 total RNA purification kit (LC Science, USA), following the manufacturer’s procedure. And equal quantity RNA from each sample of ST and SS genotype respectively were mixed into one RNA sample. Approximately 20 μg of total mixed RNAs were used to construct degradome library for ST and SS genotype, respectively. Two degradome libraries were sequenced on Illumina Hiseq2500 platform at the LC-BIO (Hangzhou, China). The CleaveLand 4.0 and the ACGT101-DEG program were used for degradome sequencing analysis [52]. TargetFinder was further used to process degradome results and facilitated miRNA target prediction [53]. The miRNA-mRNA pairs were identified according to small RNA sequencing and degradome sequencing, and the DEmiRNA-DEmRNA pairs were identified according to small RNA, degradome and transcriptome sequencing.

### Identification of candidate coherent miRNA-mRNA modules

The candidate coherent miRNA-mRNA modules were classified into two categories, including miRNAs whose abundances were significantly up-regulated or down-regulated in ST genotype, but little changed or not found in SS genotype, or remained little change or not found in ST genotype but significantly differentially expressed in SS genotype. At the same time, all target genes of miRNAs that meet the criteria should also meet the corresponding expression trends, that is, the opposite to the expression trends of miRNAs. According to this criterion, 224 candidate coherent miRNA-mRNA modules were identified in ST and SS genotype. The orthologues of target genes were identified by BLAST against the TAIR (https://www.arabidopsis.org/) and EnsemblPlants (http://plants.ensembl.org/index.html) database with the following parameter: E-value ≤ 1 × 10^–5^, identity > 50%.

### Co-expression network and functional enrichment analysis

The co-expression network was analyzed using the WGCNA package [54]. The minimum gene number of each co-expression gene module was set to 30. Cytoscape v3.8.0 was used to visualize the regulatory network of candidate genes [55]. Gene annotation and functional enrichment (GO and KEGG) were performed using KOBAS 3.0 website [56, 57]. The terms with adjust p-value (FDR) ≤ 0.05 were considered to be significantly enriched.

### Validation of mRNA and miRNA Expression by qRT-PCR

To validate the expression of the salt-responsive miRNAs and their targets, two miRNA-mRNA pairs in two genotypes were randomly selected for qRT-PCR analysis. The expression of miRNAs was detected by Poly (T) RT-PCR. In order to produce miRNA fused Poly (T) cDNA, 1 μg total RNA was used for the reverse transcription with miRNA mature sequence–specific Poly (A) RT primers according to the Poly (T) RT-PCR protocol [58]. qRT-PCR reaction was performed on a QuantStudioTM 7 Flex System (Thermo Fisher Scientific, USA) using SYBR® Green Premix Pro Taq HS qPCR Kit (Accurate Biology, China) with the following thermal cycling conditions: 95 ℃ for 30 s followed by 40 cycles of 95 ℃ for 3 s, 60 ℃ for 30 s. All reactions were performed in three biological replicates. The expression levels were calculated using the 2^−ΔΔCT^ method with *TdGADPH* as the internal reference. The primers used in this study were listed in the Additional file 1: Table S9.

### Yeast transformation, screening and annotation of wild emmer cDNA library

The total RNA from the salt-treated samples of ST genotype were used to construct the full-length cDNA library following the method described by previous study [59]. Successful transformants were selected on Luria–Bertani (LB) agar plates supplemented with 100 µg/mL ampicillin as the cDNA library. The PCR amplification products and the digested pYES2 vector were purified with cutting gel recovery. Yeastmaker ™ Yeast Transformation System 2 kit (Clontech) and *S. cerevisiae* strain (*INVSc1*) were used to performed transformation, as described by Wang et al. [59]. To identify the suitable screening concentration for the selection of transformed yeast resistant to high salinity, 100 µL of yeast work solution and control strain (transformed with empty vectors) were grown on SD-Ura plates containing 0 mM, 0.5 mM, 1.0 mM, 1.3 mM, 1.5 mM and 2.0 mM NaCl, respectively. Further, yeast work solution was cultured in SD-Ura plates with 1.5 Mm NaCl and the isolated plasmids were PCR amplified. Yeast cells containing the empty vector (*INVSc1_EV*) were used as a negative control. After the clones were grown, 200 clones were randomly selected for colony PCR and Sanger sequencing. The methods of sequencing and annotation were same to the previous study of Liu et al. [60].

## Supplementary Information

**Additional file 1:** **Table S1.** Details of identified miRNAs (conserved/known and novel). **Table S2.** Differentially expressed miRNAs of two genotypes at each treatment time point. **Table S3.** Details of target genes of differentially expressed miRNAs. **Table S4.** Differentially expressed mRNAs of two genotypes at each treatment time point. **Table S5.** Coherent miRNA-mRNA pairs of two genotypes at each treatment time point. **Table S6.** Salt tolerance-related miRNA-mRNA pairs. **Table S7.** Summary of co-expression network. **Table S8.** GO enrichment of genes co-expressed with salt tolerance-related target genes. **Table S9.** Primers used in this study.

**Additional file 2:** **FigureS1.** GO enrichment of all target genes of DEmiRNAs. **Figure S2.** GO enrichment of target genesof candidate salt tolerance-related DEmiRNA-DEmRNA pairs. CC: cell component;MF: molecular function; BP: biological process. **Figure S3.** Construction and validation of *T. dicoccoides* (ST genotype) cDNAlibrary. (a) Total RNA was isolated from the whole *T. dicoccoides* plants. (b) The results of spectrophotometermeasurement of RNA. (c) Detection of ds cDNA quality by agarosegel electrophoresis. M: Maker; 1: ds cDNA was amplified by P1-F/P4-R; 2: dscDNA was amplified by P2-F/P4-R; 3: ds cDNA was amplified by P3-F/P4-R. (d)Homogenization and purification of ds RNA detected by agarose gelelectrophoresis. (e) Detection of 24 randomly selected clones by agarose gelelectrophoresis. **FigureS4.** qRT-PCR analysis of salttolerance-related DEmiRNA-DEmRNA pairs. CT: control without NaCl treatment;NaCl: 150 mM NaCl treatment; ST: salt-tolerance genotype; SS: salt-sensitivegenotype. Significance between CT and NaCl samples were analyzed using student’s t-test (*P < 0.05, ***P < 0.001, N.S: not significant). The barsdisplay the means of miRNA or mRNA expression in the CT or NaCl samples. Theerror bars represent standard error of mean (SEM) of the three separatetechnical replicates of qRT-PCRexperiments.

## Data Availability

The datasets supporting the conclusions of this article are included within the article and its additional files. Small RNA, degradome and RNA sequencing data used in this study has been deposited into Genome Sequence Archive (GSA) database with the accession number of PRJCA009147 (https://ngdc.cncb.ac.cn/bioproject/browse/PRJCA009147), PRJCA009149 (https://ngdc.cncb.ac.cn/bioproject/browse/PRJCA009149) and PRJCA004138 (https://ngdc.cncb.ac.cn/bioproject/browse/PRJCA004138), and other data was provided in supporting information files.

## Abbreviations

microRNAs: MiRNAs
DEmiRNAs: Differentially expressed miRNAs
DEmRNAs: Differentially expressed mRNAs
GO: Gene ontology
KEGG: Kyoto encyclopedia of genes and genomes
