# Supplementary material for: Multi-omics reveals the key and specific miRNA-mRNA modules underlying salt tolerance in wild emmer wheat (Triticum dicoccoides L.)
Source: BMC Genomics. 2022 Oct 25;23:724. doi: 10.1186/s12864-022-08945-3 (PMC9597961; doi:10.1186/s12864-022-08945-3)
Supplement: Supplementary file 2 — Additional file 2: FigureS1. GO enrichment of all target genes of DEmiRNAs. Figure S2. GO enrichment of target genesof candidate salt tolerance-related DEmiRNA-DEmRNA pairs. CC: cell component;MF: molecular function; BP: biological process. Figure S3. Construction and validation of T. dicoccoides (ST genotype) cDNAlibrary. (a) Total RNA was isolated from the whole T. dicoccoides plants. (b) The results of spectrophotometermeasurement of RNA. (c) Detection of ds cDNA quality by agarosegel electrophoresis. M: Maker; 1: ds cDNA was amplified by P1-F/P4-R; 2: dscDNA was amplified by P2-F/P4-R; 3: ds cDNA was amplified by P3-F/P4-R. (d)Homogenization and purification of ds RNA detected by agarose gelelectrophoresis. (e) Detection of 24 randomly selected clones by agarose gelelectrophoresis. FigureS4. qRT-PCR analysis of salttolerance-related DEmiRNA-DEmRNA pairs. CT: control without NaCl treatment;NaCl: 150 mM NaCl treatment; ST: salt-tolerance genotype; SS: salt-sensitivegenotype. Significance between CT and NaCl samples were analyzed using student’s t-test (*P < 0.05, ***P < 0.001, N.S: not significant). The barsdisplay the means of miRNA or mRNA expression in the CT or NaCl samples. Theerror bars represent standard error of mean (SEM) of the three separatetechnical replicates of qRT-PCRexperiments. [file 12864_2022_8945_MOESM2_ESM.docx]

**Supplementary Figures**


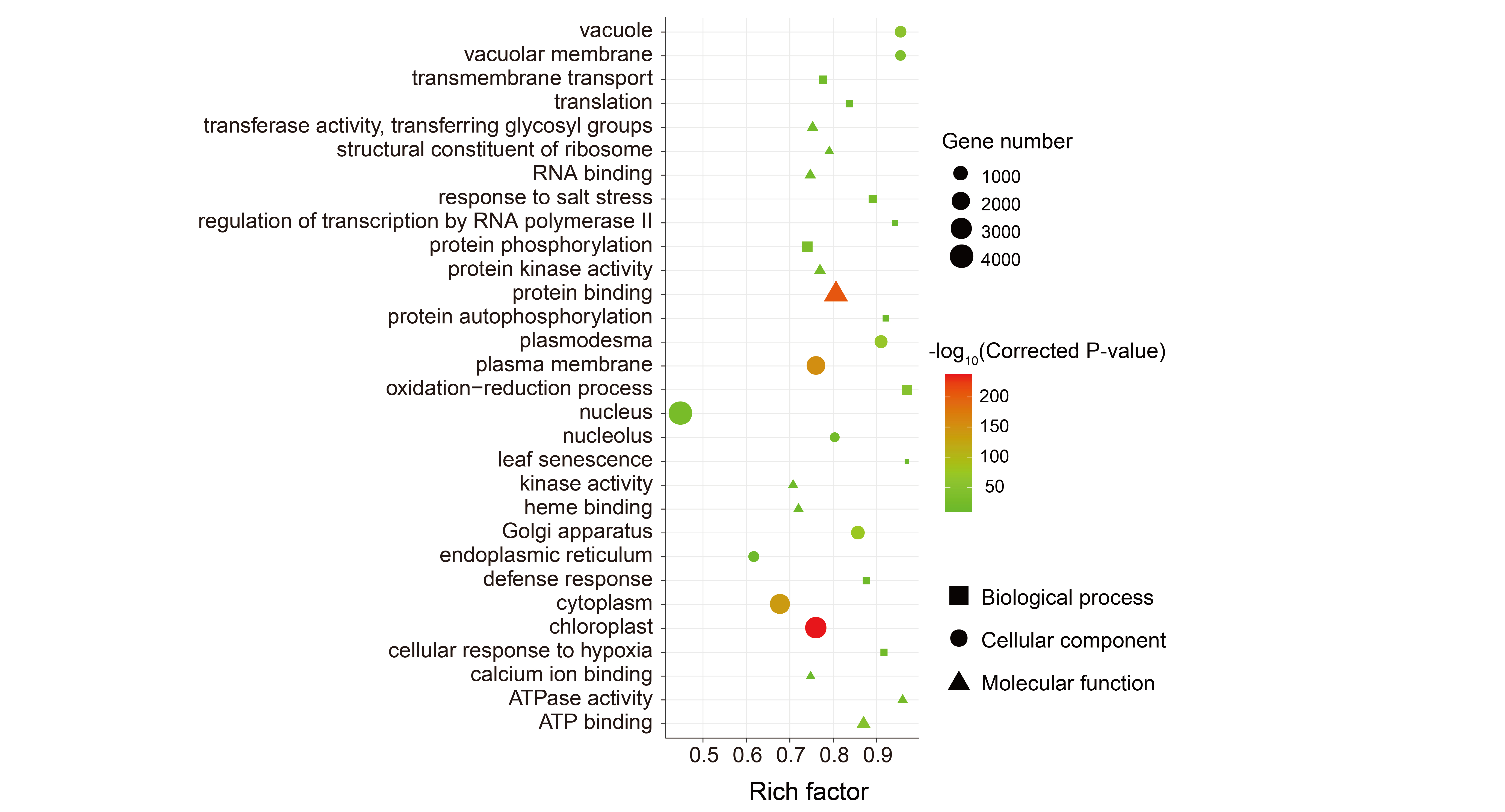


Figure S1. GO enrichment of all target genes of DEmiRNAs.


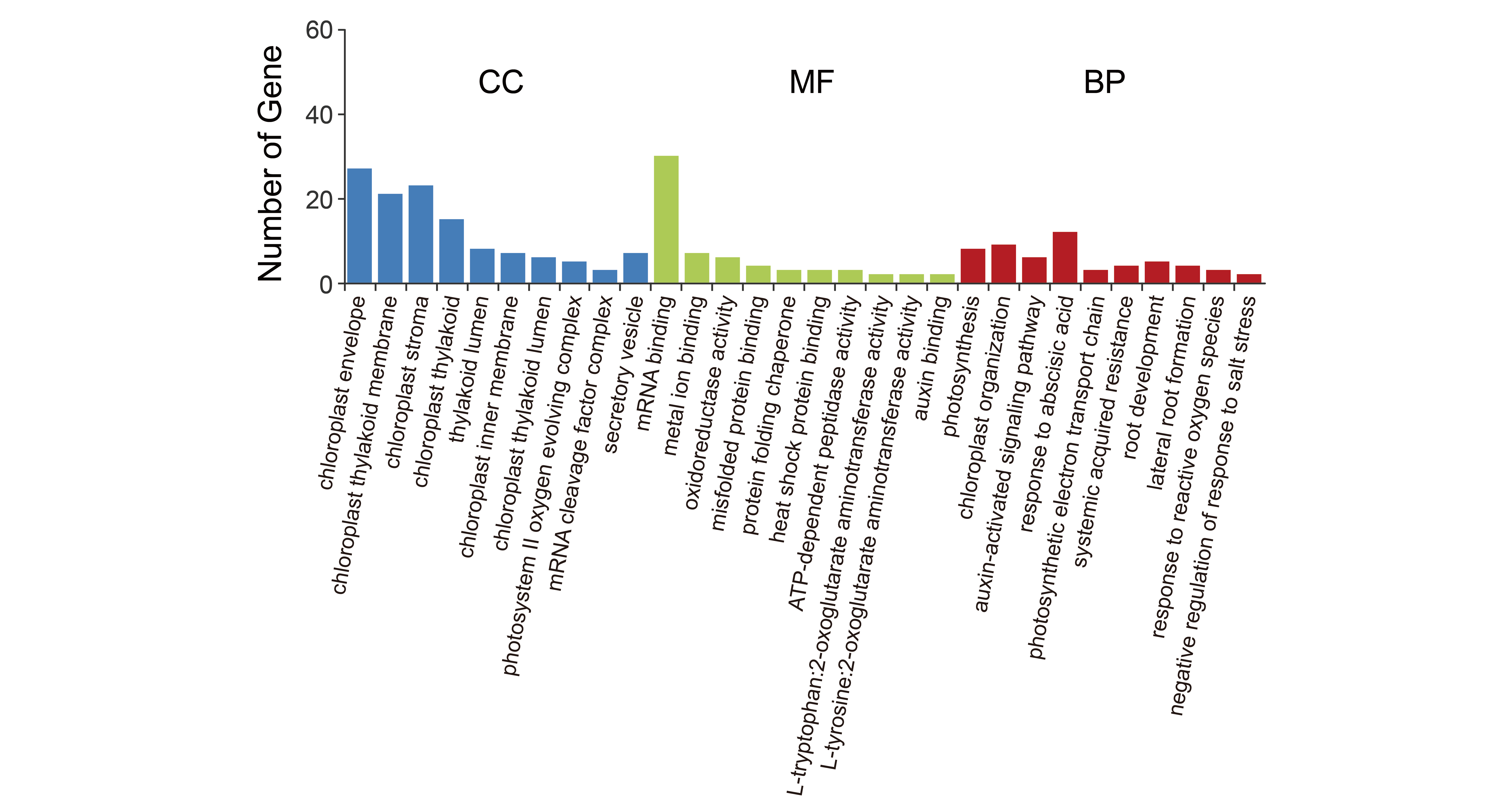


Figure S2. GO enrichment of target genes of candidate salt tolerance-related DEmiRNA-DEmRNA pairs. CC: cell component; MF: molecular function; BP: biological process.


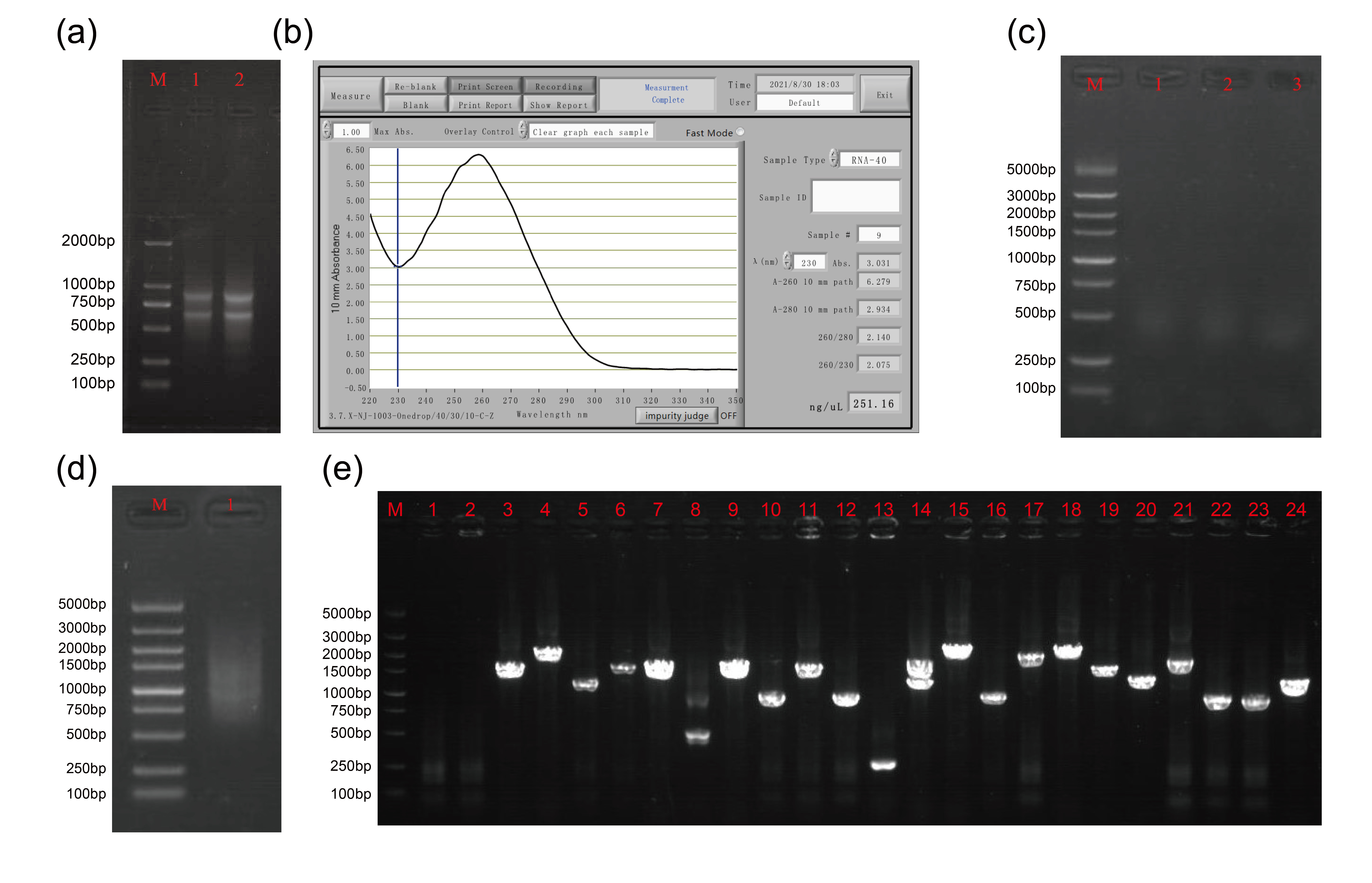


Figure S3. Construction and validation of *T. dicoccoides* (ST genotype) cDNA library. (a) Total RNA was isolated from the whole *T. dicoccoides* plants. (b) The results of spectrophotometer measurement of RNA. (c) Detection of ds cDNA quality by agarose gel electrophoresis. M: Maker; 1: ds cDNA was amplified by P1-F/P4-R; 2: ds cDNA was amplified by P2-F/P4-R; 3: ds cDNA was amplified by P3-F/P4-R. (d) Homogenization and purification of ds RNA detected by agarose gel electrophoresis. (e) Detection of 24 randomly selected clones by agarose gel electrophoresis.


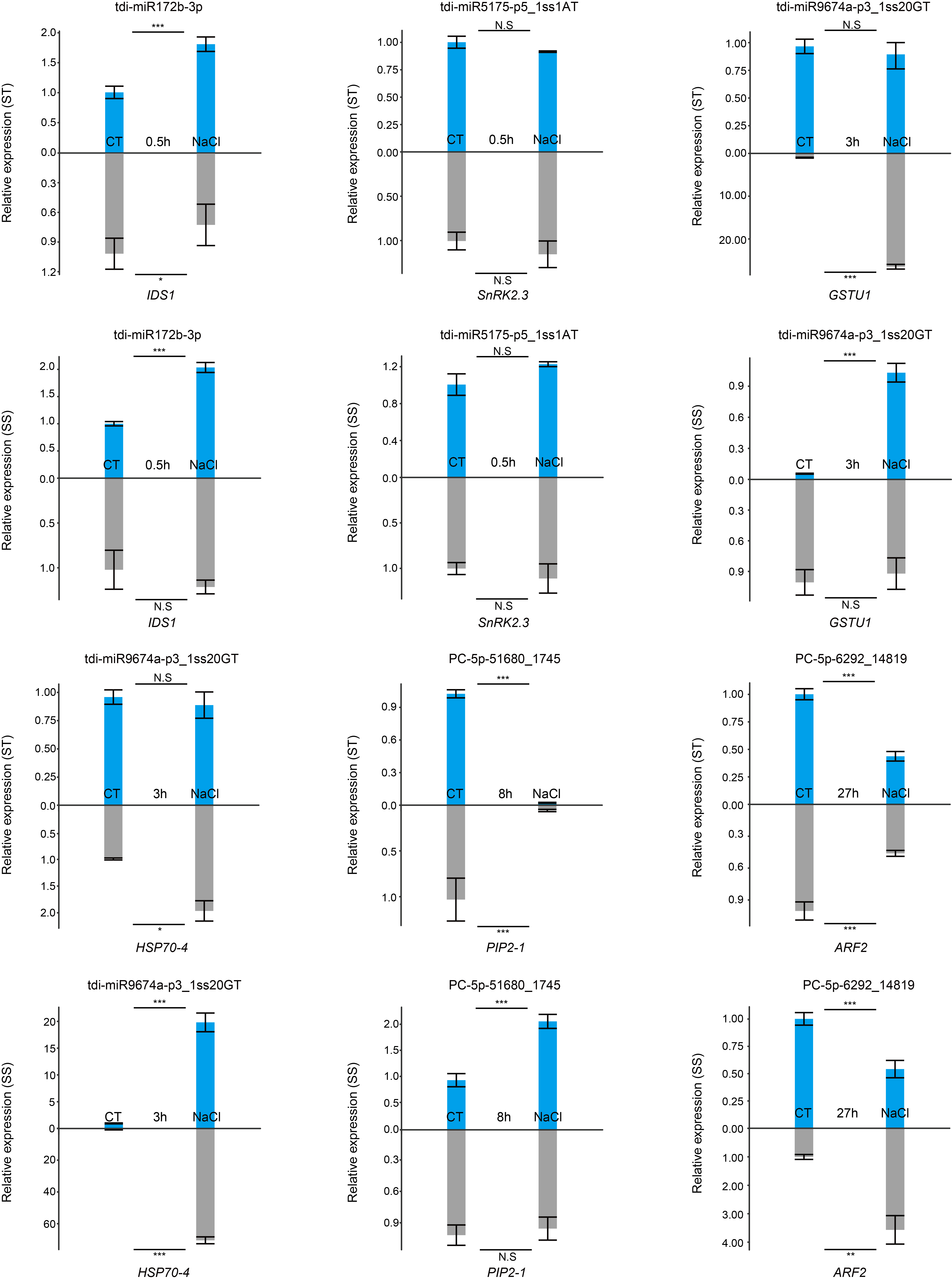


Figure S4. qRT-PCR analysis of salt tolerance-related DEmiRNA-DEmRNA pairs. CT: control without NaCl treatment; NaCl: 150 mM NaCl treatment; ST: salt-tolerance genotype; SS: salt-sensitive genotype. Significance between CT and NaCl samples were analyzed using student’ s t-test (*P < 0.05, ***P < 0.001, N.S: not significant). The bars display the means of miRNA or mRNA expression in the CT or NaCl samples. The error bars represent standard error of mean (SEM) of the three separate technical replicates of qRT-PCR experiments.
